# Absent Ureteral Efflux after Hysterectomy Leads to Diagnosis of Ureteral Atresia with Renal Atrophy

**DOI:** 10.1155/2020/9214613

**Published:** 2020-01-28

**Authors:** Olga Mutter, Ekaterina Grebenyuk, Arleen Ayala-Crespo, Kevin Yang

**Affiliations:** ^1^Department of Obstetrics, Gynecology, & Reproductive Sciences, Lewis Katz School of Medicine at Temple University, Temple University Hospital, 3401 N. Broad St, Philadelphia, PA 19140, USA; ^2^Lewis Katz School of Medicine at Temple University, Temple University Hospital, 3401 N. Broad St, Philadelphia, PA 19140, USA; ^3^Department of Urology, Lewis Katz School of Medicine at Temple University, Temple University Hospital, 3401 N. Broad St, Philadelphia, PA 19140, USA

## Abstract

Iatrogenic injury to the urinary system is a known complication of gynecologic surgery; therefore, intraoperative cystoscopy is frequently performed to assess for such injuries. However, if an abnormality is seen, the differential diagnosis extends beyond iatrogenic causes. A 42-year-old patient underwent a total abdominal hysterectomy and had absent efflux from the right ureteral orifice on cystoscopy. While iatrogenic injury was initially suspected, the intraoperative workup (including intravenous pyelography (IVP)) that ensued led to an empiric diagnosis of right ureteral atresia with ipsilateral renal atrophy that was then confirmed on postoperative imaging. When an abnormality is seen on cystoscopy following gynecologic surgery, it is important to maintain a broad differential diagnosis and to pursue an intraoperative workup with early involvement and close collaboration with urology.

## 1. Introduction

Knowledge of pelvic anatomy, including that of the urinary tract, is of paramount importance for the prevention of injury to the urinary tract at the time of gynecologic surgery. The rate of urinary tract injury during pelvic surgeries in women ranges from 0.03 to 1.7%, with hysterectomies associated with the highest rates of injury [1]. An intraoperative cystoscopy may be performed after the completion of a hysterectomy to evaluate for injury to the urinary tract. Findings that raise suspicion for a ureteral injury include decreased or lack of efflux from a ureteral orifice. These findings on cystoscopy, however, have a broad differential diagnosis that extends beyond iatrogenic ureteral injury and should include bladder atresia, ureteral atresia, ureterocele, and atrophic or dysplastic kidneys, among others. In conjunction with the standard of care, suspicious variation from normal ureteral efflux should prompt retrograde pyelographic evaluation by urology or direct exploration of the pelvis.

This case describes lack of efflux from a ureteral orifice during cystoscopy following the completion of a total abdominal hysterectomy and the intraoperative workup that ensued. This workup ultimately led to an empiric diagnosis of proximal ureteral atresia with ipsilateral renal atrophy, which was confirmed postoperatively with abdominal computed tomography imaging.

## 2. Case Presentation

A 42-year-old gravida 2 para 2002 with a history of pelvic pain and bulk symptoms likely secondary to uterine leiomyomata presented for a scheduled total abdominal hysterectomy, bilateral salpingectomy, and cystoscopy. The patient had no known history of any abnormalities of the urinary system. Her past surgical history was significant for one myomectomy and one low-transverse cesarean section with bilateral tubal ligation. Preoperative workup was pertinent for mild anemia with a hemoglobin of 11.8, a normal creatinine of 0.71, and an enlarged, leiomyomatous uterus measuring 16.6 by 6.8 by 10.6 centimeters on ultrasound. Of note, she did not require cross-sectional abdominal imaging such as a CT scan to be performed as part of her preoperative workup and had never had such imaging performed.

The procedure was performed via a midline vertical incision. Intraoperative findings were notable for an approximately 18-centimeter, nonmobile, leiomyomatous uterus and significant adhesive disease requiring extensive lysis of adhesions. Dissection included visualization of the ureters bilaterally distal to the iliac vessel bifurcation, and peristalsis was noted from the left ureter. Following hysterectomy and prior to closing the midline vertical incision, cystoscopy was performed. Cystoscopy revealed a normal bladder and left ureteral orifice with efflux of urine; however, the right ureteral orifice was not easily visualized and appeared stenotic, with no efflux. An intraoperative urology consultation was obtained for further evaluation. A cystoscopy was performed, and the bladder appeared normal with no trauma related to intra-abdominal surgery, and the left ureteral orifice was patent with efflux of urine. The right ureteral orifice, however, was difficult to discern as it appeared as a stenotic, pinpoint opening. Again, efflux of urine was not observed. Given these findings, a right retrograde pyelogram was performed and demonstrated a dilated distal right ureter. Additional injection of contrast identified extravasation of contrast proximal to the bifurcation of the iliac vessels with no opacification of the proximal ureter or renal pelvis. Additionally, urology was unable to pass a ureteral catheter proximally. A left retrograde pyelogram demonstrated normal caliber of the left ureter and renal pelvis, without distension or extravasation of contrast.

**Figure 1:**
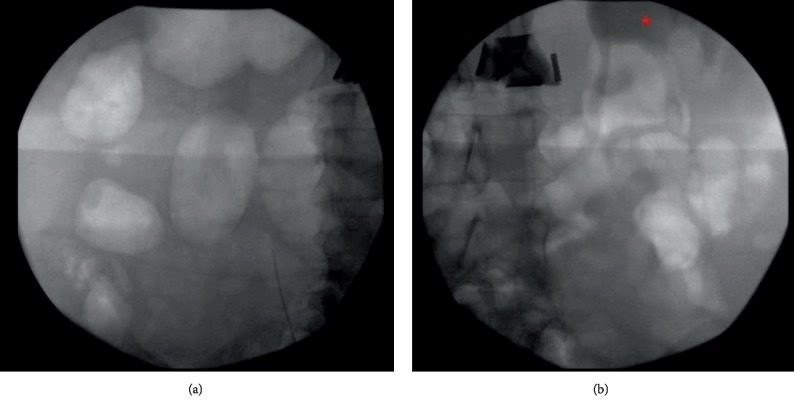
On-table intravenous pyelography demonstrating lack of opacification on the right side (a) and opacification of the renal pelvis (red asterisk) and proximal ureter on the left side (b).

Given the findings of the right retrograde pyelogram, significant suspicion for a ureteral injury was warranted. The pelvis was then explored using the same midline vertical incision, and evaluation of the right ureter was continued with retroperitoneal and abdominal dissection. The right ureter was dissected proximally to the level of the external iliac vessels and appeared to be dilated with poor tissue quality. Further proximal dissection was continued beyond the external iliac vessels in an attempt to gain ureteral length for a possible ureteral reconstruction and demonstrated that the ureteral tissue thinned out to an obliterated state at the level of the mid ureter. Of note, there was a lack of peristalsis in the right ureter throughout the entire dissection. A decision was then made to perform an intraoperative intravenous pyelogram to evaluate the bilateral renal collecting units and ureters. The left collecting system was clearly visible with adequate drainage and migration of contrast through the left ureter. In comparison, the right collecting system and ureter were devoid of any contrast pooling throughout the serial imaging (Figure 1).

At this time, given the low suspicion of injury to the right ureter and low potential for repair, a decision was made to close the case and perform additional postoperative imaging for suspected right proximal ureteral atresia and renal atrophy.

**Figure 2:**
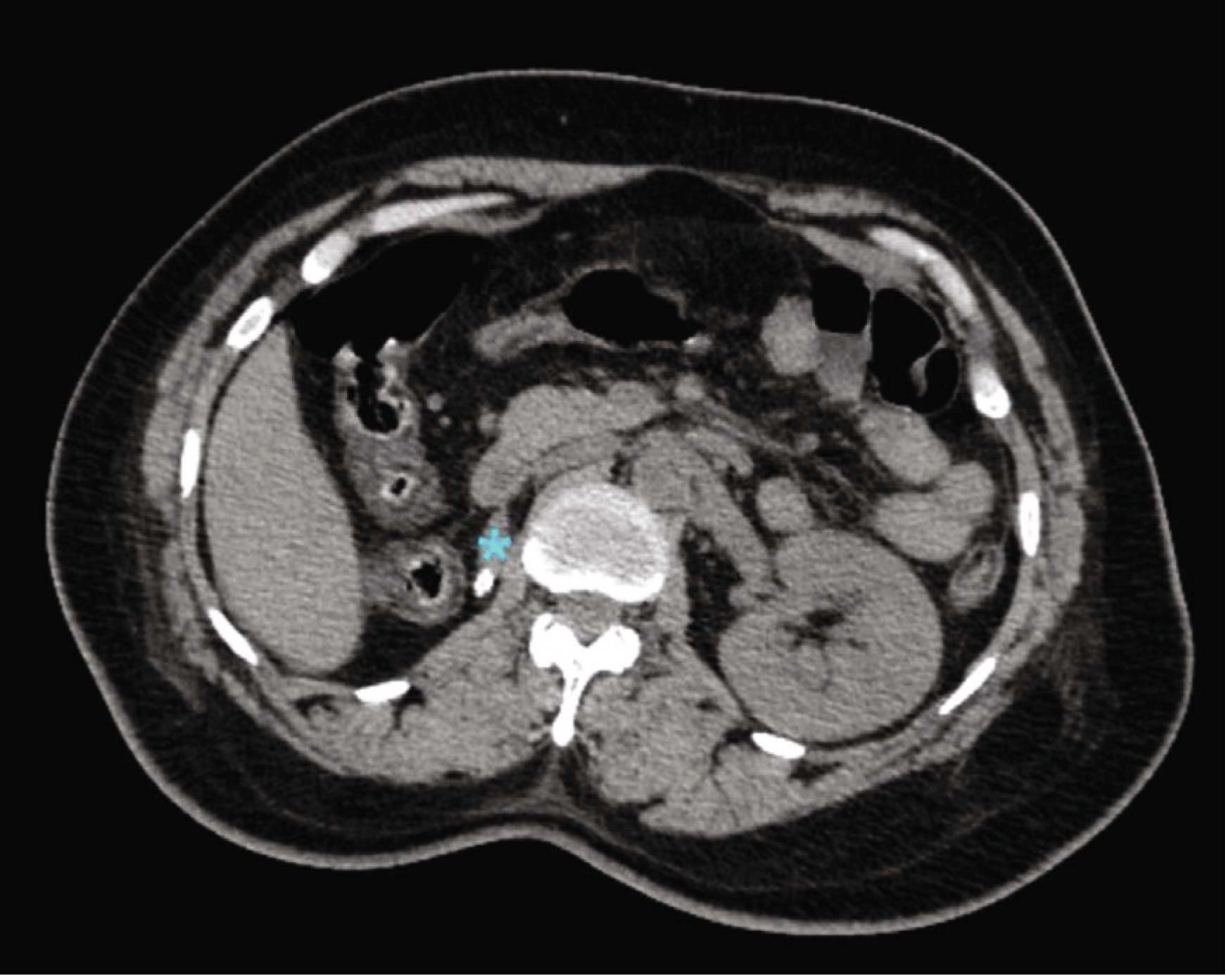
Cross-sectional imaging on postoperative day 1 showing a preexisting atrophic right kidney (blue asterisk).

On postoperative day 1, abdominal computed tomography imaging was performed and confirmed the presence of an atrophic right kidney measuring 3.4 by 2.0 centimeters with a 6-millimeter renal calculus (Figure 2). The left kidney appeared normal.

## 3. Discussion

Most adult patients who have unilateral renal agenesis or atrophy are asymptomatic and are usually diagnosed with this condition by incidental findings on abdominal imaging for unrelated medical conditions. Congenital unilateral renal agenesis, which is a relatively well-described urological anomaly, has an approximate incidence of 1 in 2000 to 2500 live births and 1 in 1000 based on autopsy results [2, 3]. This condition can occur as an isolated congenital anomaly; however, between 30 and 50% of patients have associated urological, genital, cardiovascular, gastrointestinal, and skeletal abnormalities [3]. Reproductive tract abnormalities occur in about 30% of patients with unilateral renal agenesis and occur more often in females than males, most likely due to the close association between the development of the müllerian duct with the normal development of the mesonephric duct [4, 5]. In comparison, ureteral atresia, which has been associated with atrophic or dysplastic kidney, is an extremely rare condition that has only been described through case reports [6, 7]. The patient described in this case report likely has right proximal ureteral atresia with associated ipsilateral renal atrophy. Of note, the patient had normal female reproductive tract anatomy and was able to carry two uncomplicated, full-term pregnancies.

There are two previously described cases of false-positive cystoscopic diagnosis of ureteral obstruction after hysterectomy due to a nonfunctional kidney. In these cases, unilateral nonfunctioning kidneys created similar clinical confusion at the time of cystoscopy when lack of ureteral efflux was noted [8]. As our described case is one of few very rare cases of an incidental diagnosis of a malformation of the urinary system discovered intraoperatively during a gynecologic procedure, we would like to focus our discussion on the gynecologic and urologic workup involved to arrive at the diagnosis.

When our gynecology team first noticed lack of efflux of urine from the right ureteral orifice on cystoscopy, our initial suspicion was the most common cause—iatrogenic ureteral injury—especially given the extent of the patient's adhesive disease and the extensive intraoperative dissection with lysis of adhesions. This is a well-documented complication of a hysterectomy, with the estimated rate of occurrence ranging between 0.03 and 1.7%, with the variation dependent on the type of hysterectomy [1]. Given that the findings of the retrograde pyelogram raised our suspicion for iatrogenic ureteral injury, for which the management would be surgical repair, a decision was made to perform an abdominal dissection of the ureter, using the same midline vertical incision, for direct visualization of the suspected defect. However, this dissection did not reveal a ureteral injury, but rather it demonstrated that the ureter appeared to obliterate proximally. At this point, there was a low likelihood of iatrogenic injury, and the suspicion for a malformation of the urinary system arose. An on-table intravenous pyelogram (plain film abdominopelvic radiograph after IV contrast injection) was then performed to evaluate the proximal urinary system and to appreciate a right renal unit or lack thereof.

The IVP was chosen over other imaging techniques, such as an intraoperative renal ultrasound, for a few reasons. One of the reasons for this decision was the information that an IVP versus a renal ultrasound can give such that an IVP can give complete information of the upper urinary tract from the renal pelvis to the distal ureter. In contrast, most renal ultrasounds can only evaluate the kidney or the bladder, not the course of the ureter. For this case, the renal ultrasound would in fact show whether there was a kidney present, but it would have been difficult to ascertain if the present kidney was functional and producing urine from the renal ultrasound alone. The IVP, in contrast, would not show any opacification if the kidney was nonfunctional, and we would not face that dilemma in the diagnosis. The other reason why an IVP was chosen in our case was the availability of an intraoperative renal ultrasound probe and appropriate technicians on an emergent basis at our institution. Therefore, although seemingly anachronistic and widely replaced by cross-sectional imaging in most other settings, the findings of the on-table IVP were crucial in the decision-making for this case in conjunction with the findings of the abdominal exploration, which demonstrated significant proximal ureteral atresia. The diagnosis was confirmed with a CT scan performed the next day which demonstrated an atrophic right kidney.

It is interesting to consider how this pathology evolved—is this a case of congenital proximal ureteral atresia with an atrophic kidney or a once normal kidney with silent obstruction from an obstructing ureterocele or stone, resulting in the kidney become atrophic? Based on the overall clinical evaluation, it is most likely the former. However, without previous imaging, the origin of the pathology is difficult to definitively diagnose.

A cystoscopy is performed at the conclusion of a hysterectomy to evaluate for injury to the urinary system, and iatrogenic injury is thus the first item on the differential diagnosis when an abnormality is noted on cystoscopy. However, this case demonstrates the importance of a broad differential diagnosis when a urinary tract abnormality, such as lack of efflux of urine from a ureteral orifice, is noted during gynecologic surgery. This case also highlights the importance of the IVP, which in the modern era has seen its use limited to historic teachings and emergent trauma in order to identify functional contralateral renal units before nephrectomy [9]. Most importantly, this case demonstrates the importance of early involvement and collaboration of urologic surgery with gynecologic surgery when abnormalities of the urinary system are noted intraoperatively in order to achieve the correct diagnosis and provide optimal patient care.
